# Russian-Language Mobile Apps for Reducing Alcohol Use: Systematic Search and Evaluation

**DOI:** 10.2196/31058

**Published:** 2022-01-10

**Authors:** Anna Bunova, Veronika Wiemker, Boris Gornyi, Carina Ferreira-Borges, Maria Neufeld

**Affiliations:** 1 National Research Center for Therapy and Preventive Medicine of the Ministry of Health of the Russian Federation Moscow Russian Federation; 2 Heidelberg Institute of Global Health, Medical Faculty and University Hospital Heidelberg University Heidelberg Germany; 3 WHO European Office for the Prevention and Control of Noncommunicable Diseases Moscow Russian Federation; 4 Institute for Mental Health Policy Research, Centre for Addiction and Mental Health Toronto, ON Canada; 5 Institute of Clinical Psychology and Psychotherapy Dresden University of Technology Dresden Germany

**Keywords:** alcohol, mHealth, mobile applications, screening and brief intervention, Mobile Application Rating Scale, App Behavior Change Scale, mobile phone

## Abstract

**Background:**

Personalized prevention tools such as mobile apps designed to reduce alcohol consumption are widespread in mobile app stores accessible in Russia. However, the quality and content of these mobile apps have not been systematically evaluated.

**Objective:**

This study aimed to identify Russian-language mobile apps for reducing alcohol use and to evaluate their quality and potential to change alcohol-related health behavior. It further aimed to identify apps that could facilitate screening and brief interventions in primary health care in Russia.

**Methods:**

A systematic search for mobile apps available in Russia was carried out between April 1 and 15, 2020, December 1 and 15, 2020, and in March 2021 in the iPhone App Store, Google Play Store, and the 4PDA forum. App quality was assessed using the Mobile App Rating Scale (MARS), and structured searches in electronic libraries and bibliographic databases were used to evaluate the apps’ evidence base. The number of features facilitating changes in lifestyle behavior was assessed using the App Behavior Change Scale (ABACUS).

**Results:**

We identified 63 mobile apps for reducing alcohol use. The mean MARS quality ratings were high for the subscales of functionality (3.92 out of 5, SD 0.58) and aesthetics (2.96, SD 0.76) and low for engagement (2.42, SD 0.76) and information (1.65, SD 0.60). Additional searches in electronic libraries and bibliographic databases (eLibrary, CyberLeninka, Google Scholar) yielded no studies involving the identified apps. ABACUS scores ranged from 1 to 15 out of 25, with a mean of 5 (SD 3.24). Two of the identified apps might be useful for screening and brief interventions in Russian primary health care after improvements in content and scientific testing.

**Conclusions:**

Russian-language mobile apps for reducing alcohol use are accessible in the app stores. Many of them are aesthetically pleasing, functional, and easy to use. However, information about their scientific trialing or testing is lacking. Most apps contain a low number of features that facilitate changes in lifestyle behavior. Further research should examine the context of Russian-language mobile apps for reducing alcohol use. Our findings underline the need to develop evidence-based apps to mitigate alcohol consumption in Russia and elsewhere.

**Trial Registration:**

PROSPERO International Prospective Register of Systematic Reviews CRD42020167458; https://www.crd.york.ac.uk/prospero/display_record.php?RecordID=167458

## Introduction

### Background

Alcohol is one of the leading risk factors contributing to the global burden of disease and mortality [1-3]. The World Health Organization (WHO) estimates that globally, about 3 million deaths are caused by alcohol use each year, almost 1 million of which occur in the WHO European Region as the region with the highest level of per capita alcohol consumption. Drinking alcohol contributes to the development of more than 200 diseases and injuries. It increases the risk of cardiovascular and digestive diseases, neoplasms, mental and behavioral disorders (not limited to alcohol use disorders) as well as violent crimes, suicides, and road traffic accidents [4,5]. The impact of alcohol on mortality in Russia has been well documented, and Russia remains one of the countries with the largest alcohol-attributable burden of diseases worldwide, although substantial improvements were made over the last decade [6-9]. The WHO has recently launched the SAFER initiative to reduce alcohol-related harm, which recommends that health services should provide prevention and treatment interventions to individuals and families at risk of or affected by alcohol use disorders and associated conditions [10]. One of the 5 high-impact interventions of SAFER is the screening and brief intervention (SBI) programs in primary health care (PHC) [10,11].

There is a large body of research supporting the effectiveness of SBI in reducing alcohol consumption and other alcohol-related outcomes [12-15]. Attempts to introduce SBI in the Russian PHC for patients at risk for harmful alcohol use began in 2013 when legislative changes allowed the establishment of SBI as a part of Russia’s dispanserization program within PHC facilities [16]. Dispanserization is a set of standardized measures in PHC that includes preventive medical examination for assessing the state of health and is carried out in relation to certain groups of the population in accordance with the legislation of the Russian Federation. Following the currently established provisions, dispanserization includes an evidence-based 2-step screening procedure aiming to provide early and timely detection of conditions and diseases as well as risk factors for their development, including the nonmedical use of drugs and psychotropic substances [17]. The introduced SBI as within the broader dispanserization framework consists of 2 steps [17]. In the first step, the self-administered 3-item version of the Alcohol Use Disorders Identification Test-Consumption (AUDIT-C) is used to detect patients at risk [17,18]. If the screening result is positive, that is, the score exceeds the sex-specific cut-off, the patient is asked to complete the full 10-item version of the Alcohol Use Disorders Identification Test (AUDIT) as part of an interview with a health care professional. The health care professional then provides a brief intervention depending on the results [17,19]. Despite the effectiveness of SBI, there are several barriers to their widespread implementation in PHC settings in Russia beyond the dispanserization framework [20-22]. The AUDIT is based on the concept of a standard drink, which was introduced in several countries worldwide as a measure of alcohol consumption and to provide information about alcohol consumption to consumers, which was also as part of communicating the number of standard drinks on labels of alcoholic beverages [19]. In practice, using the standard drink concept remains a challenge for PHC professionals. PHC professionals report that the concept is not understandable for patients and difficult to calculate with, especially for patients that engage in heavy episodic drinking [23,24]. Moreover, delivering a brief intervention requires specific skills and knowledge as well as additional time and resources from the PHC professionals [20,21].

The development of electronic systems to deliver or support SBIs can potentially address some of these challenges and support health care workers. For instance, electronic devices such as smartphones and tablets can be used instead of the traditional paper-and-pencil screening tests and facilitate counting standard drinks as part of the risk assessment and support the delivery of brief interventions. Moreover, electronic SBIs are also potentially more flexible and can be adapted to take into account the regional patterns of alcohol consumption and make the assessment more personalized. They can also potentially reach larger audiences beyond the health sector [25,26].

The growing popularity of mobile phones and the active development of mobile internet in all regions of Russia open up great opportunities for using mobile apps as tools to change individual health behavior [27]. Mobile apps can provide an additional resource for preventive interventions catering to at-risk populations. However, to be successful, such interventions require the health care professional to select effective, evidence-based, and field-proven mobile apps [28]. A study by Abroms and colleagues [29] showed that making such a choice is difficult since many mobile apps contain inaccuracies and low-quality information, are not tested in practice, or lack an evidence base. Abroms et al [29] point out the potential dangers of such apps, ranging from misinformation to misleading risk level estimates. A rigid evaluation of apps for reducing alcohol use is therefore of great interest to both alcohol consumers and health care professionals. While former studies have described the features of highly rated Russian-language apps for reducing alcohol use, they did not evaluate the app quality and the potential to change alcohol-related health behavior by using validated instruments [30,31]. By closing this gap and providing researchers and health care professionals with an overview of the currently available evidence-based apps for reducing alcohol use, this study may contribute to facilitating the provision of SBI programs in the Russian PHC.

### Objective

The aim of this study was to conduct a systematic search and evaluation of Russian-language mobile apps for reducing alcohol use. The specific objectives were to (1) create an overview and establish a list of relevant apps available in Russia, (2) assess their overall quality and evidence base, and (3) evaluate if any of the available apps could be used to support the provision of AUDIT-based SBI in Russia and its broader implementation in PHC facilities.

## Methods

### Study Design

The study was performed in 2 steps. In step 1, we conducted a systematic app store search to identify the apps for reducing alcohol use, following the Preferred Reporting Items for Systematic Reviews and Meta-Analyses (PRISMA) [32]. In step 2, we evaluated the identified apps by using the Mobile App Rating Scale (MARS) [33] and the App Behavior Change Scale (ABACUS) [34]. For specific steps of the rating procedure, please see below. The study protocol was published in PROSPERO (Prospective Register of Systematic Reviews) [35] under registration CRD42020167458 (review ongoing).

### Step 1: Systematic App Store Searches

#### Eligibility Criteria

We defined mobile apps for reducing alcohol use as tools for tablets or smartphones that facilitate behavioral change related to alcohol use. We excluded apps that were clearly not aimed at the reduction of alcohol use, such as games, barcode scanners as part of the Unified State Automated Information System (EGAIS) tracking alcohol distribution and sales under the Russian Federal Service for Alcohol Market Regulation [36], recipes for alcoholic drinks, and wallpapers. Only currently available and working Russian-language apps with at least a basic or trial version free of charge were included.

#### Search Strategy

Six systematic searches in the iPhone App Store, Google Play Store, and a Russian internet forum of mobile apps, that is, 4PDA [37] were conducted between April 1 and 15, 2020, December 1 and 15, 2020, and in March 2021. Two native Russian speakers conducted these 6 independent searches on different dates to account for additional mobile apps that were created during the COVID-19 pandemic. Keywords included the Russian words for “alcohol,” “alcoholic drinks,” “spirits,” “beer,” “vodka,” “drink alcohol,” “alcohol calculator,” “alcohol tracker,” “sober,” “alcohol monitoring,” and “breathalyzer” (details in PROSPERO protocol [35] and Multimedia Appendix 1) and were entered through the general search bar of the app stores and the forum.

#### Screening and Selection of Apps

In the first step, we recorded the name, app icon, developer, store, platform, brief description, and URL of all the available alcohol-related mobile apps. Next, duplicates were removed, and app store descriptions were screened against inclusion criteria. We retained only 1 record if identical versions of an app were available for Android and iPhone operating systems (iOSs). All remaining apps were downloaded onto the study devices (Samsung Galaxy Tab A 7.0 SM-T285 8GB/Android version 9, Lenovo Tablet TB-X704L 64G/Android version 7, and iPhone 11/iOS version 14.0.1). Apps that could not be opened on these devices were excluded.

#### Data Extraction

The following information was extracted for all the included apps: app name, the app’s star rating on the platform, number of installations, developer, current version, number of ratings for current version, last update, existence of a basic version and paid premium versions, and platform. All included apps were available and all data were updated in the last week of March 2021.

### Step 2: Evaluation of Mobile Apps

#### Measures/Rating Tools

We used 2 scales to rate the identified mobile apps. The MARS scale, assessing the quality of the mobile apps, contains 23 items across 5 subscales: engagement, functionality, aesthetics, information, and subjective quality [33]. Each item is rated on a 5-point scale from 1 (lowest quality) to 5 (highest quality). The overall app quality is assessed by calculating the mean scores of the first 4 subscales and the total mean score. The subjective quality score describes the raters’ personal liking of the app and should be reported separately if assessed. Subjective quality was not assessed in this study. The ABACUS scale, evaluating the apps’ potential to facilitate behavior change, contains 21 items across 4 subscales: knowledge and information, goals and planning, feedback and monitoring, and actions [34]. The total score is obtained by counting the number of items answered affirmatively. The MARS and the ABACUS showed good internal consistency and interrater reliability (MARS, α=.92; intraclass correlation coefficient [ICC]=0.85 and ABACUS, α=.93; ICC=0.92) [33,34].

A full evaluation of all the included apps was carried out by a first rater. A second rater independently evaluated a random sample of 30% (19/63) of the apps. Both raters were prepared for their task by completing a MARS video training tutorial [38]. To rate an app’s evidence base, as measured in MARS item 19, raters searched for randomized studies in the electronic libraries and bibliographic databases eLibrary, CyberLeninka, and Google Scholar by using the app’s name as a keyword as suggested by the MARS authors [38].

If the mobile app requested the input of demographic characteristics or consumption data, the following data were used: female gender, 30 years of age, body weight of 60 kg, height of 170 cm, and alcohol consumption on the last occasion as 200 ml of 40% vodka. If required, the maximum legal blood alcohol content was set to 0.3 ppm.

#### Classification of Apps and Criteria for Potential Use in SBI Programs

The identified apps were classified according to their main features. For this purpose, the following data were extracted: the app’s ability to estimate blood alcohol concentration and sobering time, its ability to record personal alcohol consumption, the presence of SBI elements, the presence of a “sobriety counter” to count the time since the last drinking occasion, and the app’s ability to support the reduction of alcohol use in a structured way. To investigate the potential of the available apps for supporting the provision of SBI in Russia, app descriptions and main features were reviewed against 2 criteria: (1) availability of AUDIT (2) whether the app provided any type of brief intervention.

#### Data Analysis

Statistical analysis and data visualization were carried out in Excel (Microsoft Excel for Office 365) and SPSS Statistics 20 (IBM Corp). Measures of interrater reliability were obtained by calculating the ICCs for all MARS and ABACUS subscales [39], using a 2-way mixed effects and average measures model with absolute agreement [40]. Descriptive analysis included total sample size, percentage, median, mean, and standard deviation.

## Results

### Systematic App Store Searches

**Figure 1:**
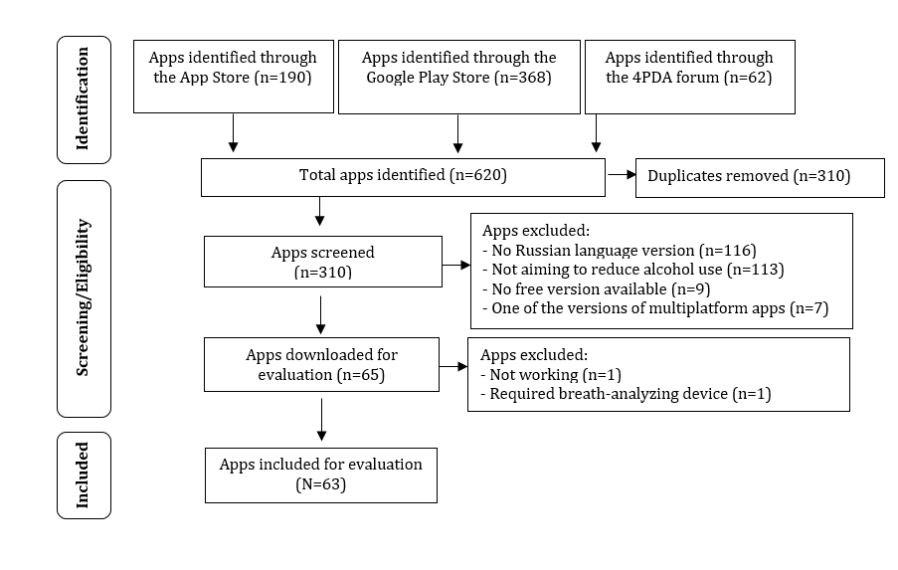
PRISMA (Preferred Reporting Items for Systematic Reviews and Meta-Analyses) flow chart of app selection.

A total of 620 alcohol-related apps were identified through keyword searches in the iOS App Store, Google Play Store, and 4PDA (Figure 1). After removing duplicates, 310 apps were screened against inclusion criteria, leaving 65 apps for reducing alcohol use for further download and evaluation. Among the downloaded apps, 2 had to be excluded as they did not work properly or required connection to a breathalyzer. Finally, 63 apps were included for evaluation, 51 of which were available only in the Google Play Store and 5 only in the iOS App Store. Only 7 apps were available in both stores (Multimedia Appendix 2).

### Overview of the Included Apps for Reducing Alcohol Use

The included apps were grouped into 6 categories according to their main feature: apps estimating blood alcohol concentration and sobering time (n=29), apps recording personal alcohol consumption (n=15), apps for SBIs (n=2), apps counting the time since the last drinking occasion (sobriety counters, n=8), apps with structured support to reduce alcohol use (n=4), and other apps for reducing alcohol use (n=5). Most apps were designed for Android systems (n=51); a minority were available for iOS (n=5) or both operation systems (n=7). A total of 19 apps were created by commercial organizations; 1 app was developed by a science center [41]. In 43 cases, no further information on the developer’s legal status could be obtained. Most of the apps (53/63, 84%) had last been updated between 2016 and 2021. A minority (n=16) offered a paid upgrade version featuring the removal of advertisements and the use of additional features. According to Google Play statistics, the median number of installations was 10,000. No comparable information was provided in the iOS App Store and 4PDA. The median star rating of all apps in Google Play and iOS App Store was 3.9, based on a median of 81 ratings. Apps with structured support to reduce alcohol consumption were downloaded most often, with a median of 300,000 installations. The median star rating of the apps in this category was 4.8, based on a median of 4418 ratings.

### Apps Estimating Blood Alcohol Concentration and Sobering Time

The main feature of this group of apps (n=29) was the estimation of the maximum blood alcohol concentration and sobering time. Most apps (n=14) were based on Widmark’s equation [42], 2 apps used Watson’s equation [43], and 13 apps provided no information about the method of calculation. Seven apps only allowed to calculate the maximum blood alcohol concentration (n=4) or sobering time (n=3). The other 22 apps provided a combination of both mentioned features (n=16) or offered additional features such as the estimation of “no-driving” time after drinking (n=10), the estimation of the maximum alcohol consumption to sober up by a certain time (n=2), a drinking diary (n=2), or the option to unlock achievements for reducing alcohol consumption (n=1).

### Apps Recording Personal Alcohol Consumption

Apps in this group (n=15) provided detailed drinking diaries (n=4), consumption calendars allowing users to indicate on what days they drunk alcohol (n=9), or both functions combined (n=2), featuring statistics of consumption per day, week, month, year. In some apps (n=4), users could calculate costs related to their alcohol use and see how much money they saved by cutting down their consumption.

### Apps for SBIs

We found only 2 Russian-language apps fulfilling our criteria to potentially facilitate SBI. Both apps allowed users to complete the AUDIT. The first app provided detailed instructions for brief interventions aimed at health care professionals and a standard drink calculator allowing users to choose consumed alcoholic drinks and calculate the number of standard drinks consumed. The second app provided information on the individual level of risk and alcohol-related harm according to AUDIT results.

### Apps Counting the Time Since the Last Drinking Occasion (Sobriety Counters)

We identified 8 apps in this group. Two of them consisted of a simple timer, counting the time since the last drinking occasion. The other 6 apps included additional motivation components such as a progress bar and achievements to be obtained (n=4) or inspiring citations (n=2). Two apps featured a chat where users could share their experiences. One app allowed users to consult with a medical specialist and to observe positive changes connected to alcohol abstinence in the physical appearance of the visualized avatars.

### Apps With Structured Support to Reduce Alcohol Use

A total of 4 apps featured structured support to help users quit drinking or to reduce their alcohol consumption. Most of these apps provided a plan with daily tasks (n=3), a sobriety counter (n=4), a drinking diary (n=2), and motivation components. Motivation components included a progress bar and achievements to be obtained (n=3), inspiring articles or citations (n=4), daily notifications (n=4), encouraging pictures or videos (n=2), and a visualization of the positive health consequences of alcohol abstinence (n=2). One app had a community chat where users shared their experience of reducing consumption or quitting alcohol. One app provided a blood alcohol concentration calculator. Three apps allowed users to complete the AUDIT (n=2) or the Michigan Alcohol Screening Test (n=1) [44].

### Other Apps for Reducing Alcohol Use

Five apps could not be assigned to any of the aforementioned categories. These included an app for audio hypnosis, an app allowing to record withdrawal symptoms, an app featuring notifications about alcohol-related harm, an app allowing to estimate the dose of consumption needed to relax, to get drunk or to have fun, and an app for counting unplanned alcohol drinking occasions after quitting drinking. 

### Behavior Change Techniques Featured in the Apps

The number of behavior change features provided by each app as reflected in ABACUS scores (Multimedia Appendix 2) ranged from 1 to 15 out of 21, with a mean of 5 points (SD 3.24). A great majority (54/63, 86%) of the apps requested individual baseline information and 71% (45/63) of the apps provided (individualized) user feedback. Many apps allowed the user to self-monitor their behavior (36/63, 57%) and customize or personalize certain app features (32/63, 51%). Table 1 shows the frequencies of the 21 behavioral change features evaluated in the apps. All ABACUS scores showed high interrater reliability (2-way mixed ICC=0.96; 95% CI 0.90-0.98).

The largest number of behavior change techniques was found in the categories of apps with structured support to reduce alcohol use and apps counting the time since the last drinking occasion (sobriety counters). Out of the 12 apps in these 2 groups, 9 apps featured more than 7 behavior change techniques. Apps estimating blood alcohol concentration and sobering time provided the lowest number of behavior change techniques, with an average ABACUS score of 2.38 (SD 1.37).

**Table 1:** Behavioral change features in the apps for reducing alcohol use (N=63).

Behavioral change feature		Apps providing the feature, n (%)
**Knowledge and information**		
	Ability to customize and personalize features	32 (51)
	Consistency with national guidelines or created with expertise	3 (5)
	Request for baseline information	54 (86)
	Instruction on how to perform the behavior	6 (10)
	Information about the consequences of continuing or discontinuing behavior	22 (35)
**Goals and planning**		
	Request for willingness for behavior change	6 (10)
	Setting of goals	7 (11)
	Ability to review goals, update, and change when necessary	6 (10)
**Feedback and monitoring**		
	Ability to quickly and easily understand the difference between current action and future goals	9 (14)
	Ability to allow the user to easily self-monitor behavior	36 (57)
	Ability to share behaviors with others or allow for social comparison	12 (19)
	Ability to give the user feedback—either from a person or automatically	45 (71)
	Ability to export data from app	4 (6)
	Material or social reward or incentive	7 (11)
	General encouragement	12 (19)
**Actions**		
	Reminders or prompts or cues for activity	13 (21)
	App encourages positive habit formation	7 (11)
	App allows or encourages for practice or rehearsal in addition to daily activities	1 (2)
	Opportunity to plan for barriers	4 (6)
	Assistance with or suggest restructuring the physical or social environment	1 (2)
	Assistance with distraction or avoidance	3 (5)

### App Quality

**Figure 2:**
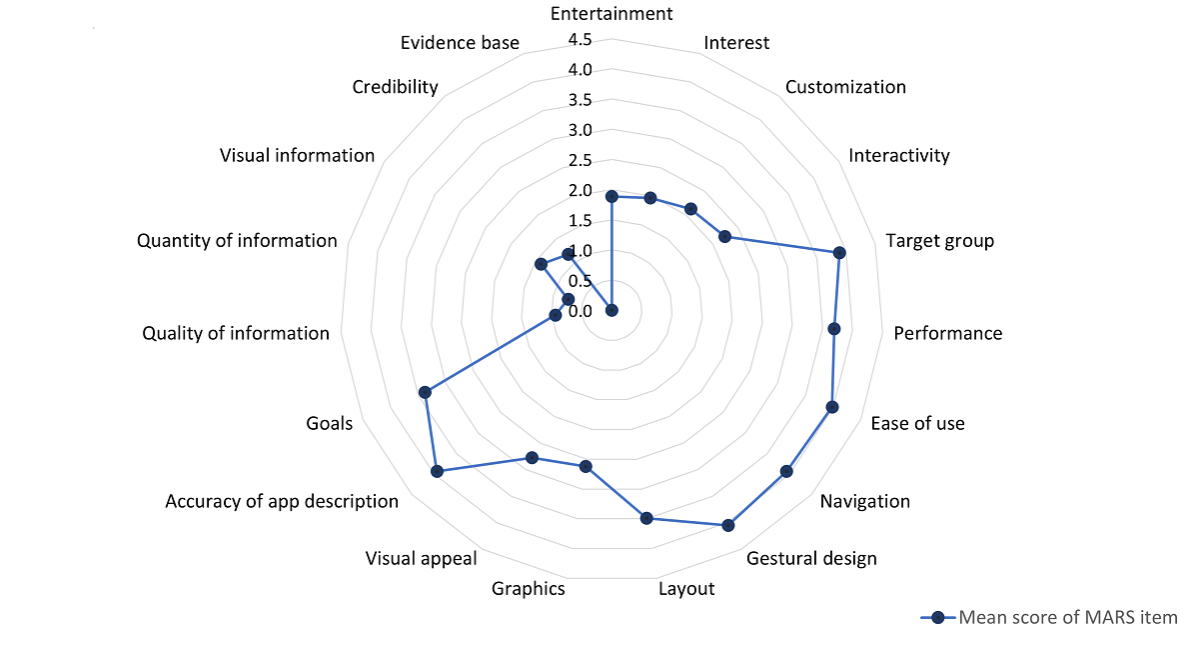
Average score of each Mobile App Rating Scale item in Russian-language apps for reducing alcohol use (N=63). MARS: Mobile App Rating Scale.

Multimedia Appendix 2 shows the subscale and overall MARS scores of all evaluated apps for reducing alcohol use. Interrater reliability was high (2-way mixed ICC=0.96; 95% CI 0.91-0.98). The average overall MARS score of all reviewed apps was 2.74 (SD 0.52). MARS item 19 “evidence base” reached the lowest mean score (0.00)—no information about scientific trialing or testing of the identified app could be obtained. Other low mean scores included quantity of information (0.75, SD 1.24), quality of information (0.94, SD 1.47), credibility (1.17, SD 0.58), visual information (1.40, SD 1.75), interest (1.97, SD 0.97), and entertainment (1.89, SD 0.99). The highest scores were obtained for gestural design (4.05, SD 0.63), ease of use (3.98, SD 0.66), accuracy of app description (3.94, SD 0.56), and target group (3.89, SD 0.84). The average score of all MARS items is shown in Figure 2. A total of 30% (19/63) of the evaluated apps reached an overall MARS score of ≥3.0. The categories of apps with strong support to reduce alcohol use and apps for SBIs reached the highest overall scores (3.74 [SD 0.18] and 3.42 [SD 0.32], respectively).

### App Potential for SBI in the Russian PHC

Out of the 63 identified mobile apps for reducing alcohol use, 4 apps contained the AUDIT, which is widely used in Russian PHC SBI. However, only 2 apps contained additional SBI elements and thus fulfilled both selection criteria. The 2 apps providing only the AUDIT (“I do not drink!” and “Sober One”) contained obvious errors. The *I do not drink!* app has a “urban” translation into Russian and contains only 9 out of the 10 AUDIT questions. The *Sober One* app is potentially more attractive for SBI as it provides a brief risk assessment. However, 1 standard dose is determined as 13.7 g of pure alcohol, which does not correspond to the value officially used in Russia [17,45]. Out of the 2 apps fulfilling both selection criteria (AUDIT and Alcoholism test), the *AUDIT* app applies the SBI algorithms from the official Russian guidelines [17]. The second app, *Alcoholism test*, evaluates the individual health risk and provides additional information about the harm associated with alcohol consumption, which may motivate a conversation with the patient. However, we did not find any information on the scientific testing or trialing of the identified apps.

## Discussion

### Principal Findings

This study is the first systematic search and evaluation of Russian-language mobile apps for reducing alcohol use in Russia. We identified and assessed 63 eligible apps, 2 of which could potentially be used in SBIs in Russian PHC facilities after improvements in content and scientific testing. The MARS app quality scores of the evaluated apps showed good functionality, aesthetics, and ease of use. However, there is ample room for improvement, especially in the area of scientific support and evidence base; no information on scientific trialing or testing of any of the apps could be obtained. Further, ABACUS scores indicated that most apps provide only few features to facilitate human behavior change, casting doubt on their effectiveness to change alcohol consumption habits. These weaknesses seem to be common not only in Russian-language apps but in comparable apps worldwide. In a recent Australian study using both MARS and ABACUS, English-language apps for reducing alcohol use obtained similar ratings as the apps evaluated in this study [46].

The analysis of the apps’ main features revealed some specific weaknesses and strong points of different app categories. Apps for calculating blood alcohol concentration and sobering time mostly used the Widmark formula developed in 1932 [42]. There are studies suggesting that this formula considerably underestimates blood alcohol concentration [47,48]. Most apps recording personal alcohol consumption and apps with structured support to reduce alcohol use featured infographics, allowing the user to quickly and visually evaluate their alcohol consumption. Some of these apps featured chats and goal-setting functions, which had an additional supportive effect. Virtual avatars with changing appearance added an element of gamification to some apps and allowed for a competitive effect between users. It is also worth highlighting the group of mobile apps with structured support to reduce alcohol use, which contained a higher number of features that facilitate changes in alcohol-related health behavior. In the future, it might be worthwhile to conduct an additional detailed analysis of these apps’ content and long-term effectiveness. Users of apps for reducing alcohol use are often not aware that most apps contain unreliable, non–peer-reviewed content, which might be noneffective or even put users at risk [29,49,50]. Currently, there is no information about apps’ evidence base or scientific background available in the App Store and the Google Play Store [50].

Some international websites such as iMedicalApps offer expert comments and reviews of medical apps to patients and health care professionals [51]. Unfortunately, none of these sites are available in Russian language. SBI for alcohol consumption is not yet broadly established and implemented at the level of PHC in Russia, although decisive action was taken to change this in the past 8 years [52]. Electronic SBIs might offer greater flexibility, and depending on their mode of implementation, potentially more anonymity to avoid stigma for PHC patients [26]. Their wide availability in the App Store and, more importantly, Google Play Store as the most popular marketplace for mobile apps in Russia, may offer new opportunities to expand personalized medical care for people with alcohol-related problems [53]. The use of mobile apps to facilitate the assessment of alcohol intake as well as the level of according risk is a promising approach and requires further study, especially in a country like the Russian Federation that is committed to implement SBIs as a routine procedure.

### Limitations

Searches were carried out in the iOS App Store, Google Play Store, and the 4PDA forum. These stores regularly update their content, meaning that mobile apps may become unavailable over time. Furthermore, search options such as language and region settings affect the selection and order of results, thereby reducing the reproducibility of the searches. App contents were not analyzed in detail nor did we assess the apps’ potential to change human behavior in the long term. This assessment may represent an area for future research.

### Conclusions

This study provides a structured overview of the main features, quality, and potential to change the alcohol-related health behavior of Russian-language apps for reducing alcohol use currently available in Russia. This overview can be used as a reference by alcohol consumers and health care professionals alike when choosing an app to facilitate the reduction of alcohol use. Although Russian-language apps for reducing alcohol use were found to be aesthetically pleasing, functional, and easy to use, most apps contained a low number of features that facilitate changes in lifestyle behavior and lacked information about scientific trialing or testing. Only 2 identified apps contained the AUDIT and additional brief intervention elements and could thus potentially be used for SBI in the Russian PHC after rigorous scientific evaluation of their effectiveness. Overall, our findings underline the need to develop evidence-based apps to mitigate alcohol consumption in Russia and elsewhere.

## Appendix

Multimedia Appendix 1 PROSPERO (Prospective Register of Systematic Reviews) protocol.

Multimedia Appendix 2 Russian-language mobile apps for reducing alcohol use.

## Abbreviations

ABACUS: App Behavior Change Scale
AUDIT: Alcohol Use Disorders Identification Test
AUDIT-C: Alcohol Use Disorders Identification Test-Consumption
ICC: intraclass correlation coefficient
iOS: iPhone operating system
MARS: Mobile App Rating Scale
PHC: primary health care
PROSPERO: Prospective Register of Systematic Reviews
SBI: screening and brief intervention
WHO: World Health Organization
