# Supplementary material for: Russian-Language Mobile Apps for Reducing Alcohol Use: Systematic Search and Evaluation
Source: JMIR Mhealth Uhealth. 2022 Jan 10;10(1):e31058. doi: 10.2196/31058 (PMC8787655; doi:10.2196/31058)
Supplement: Multimedia Appendix 1 [file mhealth_v10i1e31058_app1.docx]

## Appendix 1. PROSPERO protocol

**1.Review title**

Russian-language mobile applications for reducing alcohol use: a systematic search and evaluation

**2.Original language title**

-

**3.Anticipated or actual start date**

01.11.2020

**4.Anticipated completion date**

30.11.2020

**5.Stage of review at time of this submission**

Preliminary searches - Completed

Piloting of the study selection process - Completed

Formal screening of search results against eligibility criteria - Completed

Data extraction - Completed

Risk of bias (quality) assessment - Completed

Data analysis – Completed

**6. Named contact**

Anna Bunova

**7. Named contact email**

[Asbunova@gmail.com](mailto:Asbunova@gmail.com)

**8. Named contact address**

Petroverigskiy Pereulok 10, 101990, Moscow, Russian Federation

**9. Named contact phone number**

-

**10. Organisational affiliation of the review**

National Medical Research Center for Therapy and Preventive Medicine of the Ministry of Healthcare of the Russian Federation

Organisation web address: https://www.gnicpm.ru/

**11. Review team members and their organisational affiliations.**

Anna Bunova, National Medical Research Center for Therapy and Preventive Medicine of the Ministry of Healthcare of the Russian Federation, [asbunova@gmail.com](mailto:asbunova@gmail.com)

Maria Neufeld, WHO European Office for Prevention and Control of Noncommunicable Diseases, Moscow, Russian Federation [neufeld.maria@googlemail.com](mailto:neufeld.maria@googlemail.com)

Boris Gornyi, National Medical Research Center for Therapy and Preventive Medicine of the Ministry of Healthcare of the Russian Federation, [BGornyy@gnicpm.ru](mailto:BGornyy@gnicpm.ru)

**12. Funding sources/sponsors**

None

**13. Conflicts of interest**

None

**14. Collaborators**

Veronika Wiemker, Heidelberg Institute of Global Health, Medical Faculty and University Hospital, Heidelberg University, Heidelberg, Germany

Carina Ferreira-Borges, WHO European Office for Prevention and Control of Noncommunicable Diseases, Moscow, Russian Federation ferreiraborgesc@who.int

**15. Review question**

1. How many Russian-language mobile applications aimed at reducing alcohol use exist?

2. What is the overall quality of the existing Russian-language mobile applications for reducing alcohol use when rated with standardized tools, which measures evidence-based principles, usability, quality of design etc.?

3. Are there any Russian-language applications available for reducing alcohol use as part of a health behavior change that are free of charge, are evidence-based and are user-friendly and that can be used by healthcare professionals in the Russian Federation as a practical aid to deliver screening and brief interventions for alcohol?

**16. Searches**

Searches will be conducted by two native Russian speakers independently between 1 and 15 April and 1 and 15 December 2020 to account for any additional mobile applications for reducing alcohol intake that might have occurred during the COVID-19 pandemic. The search will cover mobile app stores App Store and Google Play and the mobile apps forum 4PDA. The search will be restricted by language and geographically and only applications available in Russian language in app stores of the Russian Federation will be included. The search will not be limited by time and all apps for reducing alcohol use will be included regardless of the date of their last update.

**17. URL to search strategy**

**Search Strategy**

**Russian-language databases/platforms that will be searched:**

*Mobile App Stores:*

- App Store (https://www.apple.com/ru/app-store/)
- Google Play (<https://play.google.com/store;?hl=ru>)

**Additionally:** *Forum of mobile apps* 4PDA (<https://4pda.ru/>)

**Keywords**

The keywords used will be in Cyrillic letters of the Russian alphabet (e.g. алкоголь). Combinations of keywords will be used, but no specific algorithm can be prepared because the platforms do not allow for specific search commands.

*Keywords for mobile apps store and forum of mobile apps*

- “алкоголь”
- “алкогольные напитки”
- “спиртное”
- “спиртные напитки”
- “пиво”
- “водка”
- “вино”
- “пить алкоголь”
- “перестать пить”
- “контролировать потребление алкоголя”
- “снизить потребление алкоголя”
- “уменьшить потребления алкоголя”
- “отследить потребление алкоголя”

**Search dates**

Searches will be conducted by two native Russian speakers independently between 1 and 15 April and 1 and 15 December 2020 to account for any additional mobile applications for reducing alcohol intake that might have occurred during the COVID-19 pandemic.

**Restrictions**

The search will be restricted geographically and by language, and we will include only Russian language apps available in app stores of the Russian Federation. The search for apps will not be limited by time. We will include all apps for reducing alcohol use regardless of the date of their last update, developer and target audience.

**18. Condition or domain being studied**

The growing popularity of mobile phones and the active development of mobile Internet worldwide open up new opportunities for using mobile applications as tools for changing health behaviors, including smoking cessation and the reduction of alcohol consumption.

This includes the growing use of digital assessment tools and the delivery of short behavioral interventions through mobile applications. Digital alcohol assessment tools are defined as tools that use an electronic display device (a personal computer, laptop or a mobile device such as a smartphone) to facilitate the quantification of the individual’s alcohol consumption. Mobile applications can be used for individual assessment of alcohol use, knowledge sharing and behavioral intention formation as well as self-motivation for actual behavior change, but also as an additional resource in the healthcare system for preventive services in the area of risk factors and lifestyles. Here, mobile apps can be used to assess individual’s drinking levels and patterns and assist healthcare professionals in assessing individual risk and provide tailored interventions. Therefore, the use is mobile apps can be a powerful tool in delivering screening and brief interventions for alcohol in healthcare setting and also beyond.

The provisions of screening and brief interventions for alcohol is not yet broadly established and implemented at the level of primary healthcare in the Russian Federation, although decisive action was taken to change this in the past five years. The use of mobile applications to facilitate the assessment of alcohol intake as well as the level of according risk and the interventions needed is a promising but yet unexplored tool in this regard.

The present contributions aims at exploring and understanding which mobile applications exist in the Russian Federation that are evidence-based, user-friendly and that can be used by healthcare professionals as practical aid to deliver screening and brief interventions for alcohol within the healthcare system.

**19. Participants/population**

*Inclusion criteria*

In the first step, we will screen all available alcohol-related mobile apps. In a second step, we will include only mobile apps aimed at behavioral change for reducing alcohol consumption.

*Exclusion criteria:*

- Mobile apps that do not aim at reducing alcohol use as a behavioral change, for instance entertainment apps, games, barcode scanners as part of the EGAIS tracking system and other purposes, recipes for alcoholic drinks, alcohol calculators that are not aimed at reduction of use (e.g. alcohol calculators aimed at quantifying the alcohol content of homemade beveragaes), wallpapers, gadget add-ons and other features that are not aimed at reduction of alcohol use.
- Apps not available in Russian language
- Apps that cannot be opened and interacted with in the Russia
- Paid versions of the app if the app has a free trial version with the relevant features
- One of the versions of apps developed for two systems iOS and Android

**20. Intervention(s), exposure(s)**

Not applicable

**21. Comparator(s)/control**

Not applicable

**22. Types of study to be included**

In the first step, all available alcohol-related mobile apps that are in Russian language and available in the Russian Federation will be included in a preliminary database. In the second step, the apps will be used by two researchers independently against the outlined exclusion criteria and only mobile apps for reducing alcohol consumption as a behavioral change target will be included in the second database. Any differences in the screening procedure and the subsequent decisions will be discussed in the larger group, involving at least two more collaborators and a consensus should be reached via discussion.

All the applications that were included in the second step will undergo a formal rating procedure.

**23. Context**

Traditionally, prevention of alcohol use disorders in the Russian Federation is the area of the specialized sub-discipline of psychiatry, known as “narcology” or addiction medicine. Prevention, treatment (including detoxification procedures), and rehabilitation of substance use disorders, are carried out in the specialized system of narcology, following the established by protocols of the Ministry of Health, within government-run and private-run in-patient and out-patient facilities. However, the narcological system does not offer any interventions for individuals with risky patterns and/or levels of substance use that do not fulfill the clinical criteria of the Russian abridged version of the 10th revision of the International Statistical Classification of Diseases and Related Health Problems (ICD-10), and these individuals do not consider narcology as an option due to various barriers that exist, including “narcological monitoring” requirements that are imposed once an individual is seeking narcological treatment. Starting from 2011, changes were incrementally made to the legislative framework that regulates the provision of medical help in Russia in order to provide preventive interventions for individuals who are at risk but do not (yet) meet any clinical criteria for substance use disorders and who might benefit from brief interventions at the level of primary healthcare (PHC). In 2013 the legislative changes have introduced specific evidence-based screening procedures for risk factors as part of a two-step screening process as part of dispanserization^[[1]](#footnote-1)^ procedures, which also includes screening for the risky use of alcohol. For detecting the “risk of harmful alcohol use” different screening tools where introduced over the years, but for now, the routine screening procedures are still limited to the dispanserization process, although they are planned to be extended over other health settings, first of all primary healthcare, in the nearest future.

As part of this development, it seems important and timely to explore the potential of mobile applications in aiding healthcare professionals among many different health settings, but primarily in PHC.

**24. Main outcome(s)**

The main aim of the present search for existing Russian-language mobile applications to reduce alcohol intake is to 1) highlight which applications exist and are available in the Russian Federation and 2) what their overall quality and evidence base are, and 3) if any of these available applications are useful in supporting the provisions of screening and brief interventions in Russia.

Measures of effect - Not applicable

**25. Additional outcome(s)**

An additional and important outcome will be a preliminary database of all alcohol-related mobile applications, which will be formed in the first step of the search procedure before including only applications that are aimed at alcohol reduction as a behavioral change. The formation of such a first broad database might give important insight into the area of digital availability and digital marketing in the Russian Federation, which are important topics in the current COVID-19 pandemic and might provide guidance on where additional regulation and/or enforcement is needed at the moment as well as in the future.

Additionally, the search will help to identify what should and needs to be considered when developing mobile applications for alcohol to help PHC healthcare workers with the provisions of screening and brief interventions.

Measures of effect - Not applicable

**26. Data extraction (selection and coding)**

Data will be extracted by two researchers independently, following a two-step procedure. In the first step, all mobile applications will be searched in Google Play, then in the App store, and finally 4PDA, using the established key words. Every application that relates to the topic of alcohol will be included in an initial excel file, noting the name of the application, the developer and a short app summary as provided on the platform only, and the two excel files of the two researchers will be merged, excluding duplicates and discussing any differences that might occur.

In the next step, the excel file will be screened against the exclusion criteria and apps that are not aimed at the reduction of alcohol use following their own description will be excluded from the file. The extraction procedure will be carried out by two independent researchers again and any occurring differences will be discussed in a broader group, involving at least two more collaborators and a case-by-case examination in order to reach consensus.

In the third step, two researchers will download the identified mobile apps from the merged and agreed list on their smartphones and start a more through data extraction. The following information will be extracted as part of this step - the app name, category, app’s rating on the platform, number of installations, developer, version, number of ratings for version, last update, the existence of cost-basic version and cost-upgrade versions, platform, brief description and focus (i.e. what the app targets), the theoretical background/strategies, affiliations, age groups, and technical aspects of the app (if available). The data for all apps will be extracted on the same week and merged within one database as part of a group discussion to ensure for consistency of the different versions of the apps available on a set qualifying date.

The final extraction list will be further used for formal rating procedures.

**27. Risk of bias (quality) assessment**

To reduce the risk of rater bias, two researchers will perform the search, the screening and the inclusion tasks independently. These processes will be accompanied by a broader group discussion and additional random checks and searches by at least one additional collaborator.

**28. Strategy for data synthesis**

The strategy for data synthesis will follow the outlined multi-step process and the final list of mobile applications that are aimed at reducing alcohol used will undergo a formal rating process of three independent raters, using standardized scales, namely the Mobile App Rating Scale (MARS)and the App Behavior Change Scale (ABACUS).

**29. Analysis of subgroups or subsets**

The initial database of all alcohol-related applications will be grouped into categories for further mapping and deeper analysis, wherever appropriate, to document the landscape of existing alcohol applications in Russia. This will allow for further insights into digital marketing techniques of alcohol, digital availability of alcohol as well as other relevant dimensions, such as online offers of narcological services as well as other interventions.

**30. Type and method of review**

Type of review – diagnostic, prevention

Health area of the review – alcohol/substance misuse/abuse, public health

**31. Language**

Russian

**32. Country**

Russian Federation

**33. Other registration details**

National Medical Research Center for Therapy and Preventive Medicine of the Ministry of Healthcare of the Russian Federation

**34. Reference and/or URL for published protocol**

Not applicable

Do not make this file publicly available until the review is complete

**35. Dissemination plans**

Do you intend to publish the review on completion? – Yes

The results of the study will be published either in a public health journal or in mHealth journal, depending on the actual outcomes of the searches.

**36. Keywords**

alcohol; mHealth; mobile applications; Russia;

**37. Details of any existing review of the same topic by the same authors**

-

**38. Current review status**

Completed but not published

Please provide anticipated publication date - 2021

**39. Any additional information**

This is the first systematic search and evaluation of Russian-language mobile applications for reducing alcohol use

**40. Details of final report/publication(s) or preprints if available**

**-**

1. In the Russian Federation, dispanserization involves regular preventive check-up activities that include preventive and specialized medical examinations for early detection and prevention of diseases at the population level. Dispanserization activities are organized within PHC facilities and follow specific pre-defined schedules for different population age groups. [↑](#footnote-ref-1)
