# Supplementary material for: Russian-Language Mobile Apps for Reducing Alcohol Use: Systematic Search and Evaluation
Source: JMIR Mhealth Uhealth. 2022 Jan 10;10(1):e31058. doi: 10.2196/31058 (PMC8787655; doi:10.2196/31058)
Supplement: Multimedia Appendix 2 [file mhealth_v10i1e31058_app2.docx]

## Appendix 2. Russian-language mobile apps for reducing alcohol use

| **App Icon** | **App Name** | **Platform** | **Rating** | **Number of installations** | **Developer** | **Number of ratings** | **Last update** | **Version** | **Cost - basic version** | **Cost - upgrade version** | **MARS** | | | | | **ABACUS** |
| --- | --- | --- | --- | --- | --- | --- | --- | --- | --- | --- | --- | --- | --- | --- | --- | --- |
|  |  |  |  |  |  |  |  |  |  |  | **A** | **B** | **C** | **D** | **App quality mean score** |  |
| **Mobile apps for estimating blood alcohol concentration and sobering time** | | | | | | | | | | | | | | | | |
| 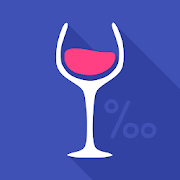 | Alkometr | Android | 4.4 | 100000+ | Ruslan Valiahmetov | 1617 | 18.10.2019 | 2.5.2 | Free | N—A | 2.20 | 4.00 | 3.00 | 2.14 | 2.84 | 5 |
| 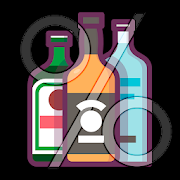 | Alcohol calculator | Android | 3.9 | 10000+ | SonnikSleep | 236 | 21.04.2020 | 14.0 | Free | N—A | 1.60 | 5.00 | 3.33 | 1.71 | 2.91 | 2 |
| 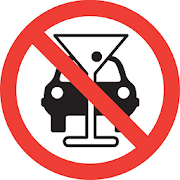 | Alcotester Alco Calc | Android | 3.2 | 10000+ | yuribond | 26 | 22.06.2019 | 2.0.9 | Free | N—A | 1.60 | 3.75 | 2.00 | 1.29 | 2.16 | 3 |
| 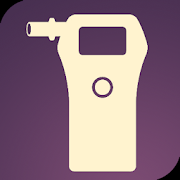 | Alcotester | Android | 2.8 | 10000+ | Diamond Software Team | 12 | 07.05.2019 | 1.3 | Free | N—A | 1.60 | 4.00 | 2.67 | 1.14 | 2.35 | 2 |
| 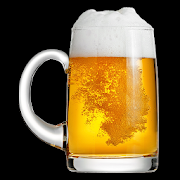 | Calculator of alcohol | Android | 3.6 | 50000+ | App Sega | 81 | 03.03.2018 | 1.0 | Free | N—A | 1.40 | 3.75 | 2.67 | 1.14 | 2.24 | 2 |
| 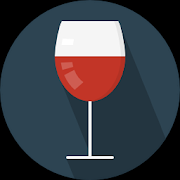 | AlcoTime - alcotester | Android | 3.9 | 10000+ | Articat | 50 | 01.10.2017 | 3 | Free | 1$ | 2.00 | 3.50 | 2.33 | 2.43 | 2.57 | 3 |
| 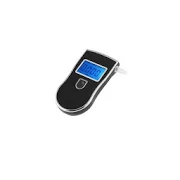 | Alcohol calculator | Android | 3.9 | 50000+ | AF mobile | 607 | 14.07.2016 | 2.6 | Free | N—A | 2.00 | 4.75 | 1.67 | 1.29 | 2.43 | 2 |
| 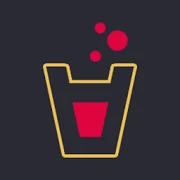 | AlcoExpert: | Android | 3.9 | 1000+ | Belonica | 7 | 17.02.2019 | 1.0.2 | Free | N—A | 3.00 | 5.00 | 3.67 | 1.57 | 3.31 | 3 |
| 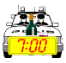 | Alcohol calculator. | Android | 3.7 | 1000+ | Maksim Gusev | 6 | 01.12.2017 | 1.0.0 | Free | N—A | 1.60 | 3.25 | 2.33 | 1.57 | 2.19 | 2 |
| 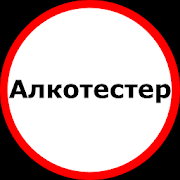 | Alkotester | Android | 3 | 10000+ | Dmitry Tulupov | 31 | 26.01.2016 | 2 | Free | N—A | 1.60 | 3.25 | 2.33 | 1.00 | 2.05 | 1 |
| 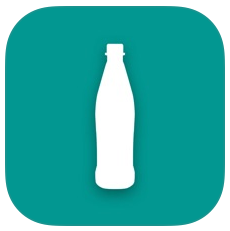 | Alkometr | Android | 3.1 | 10000+ | IDS BORJOMI | 40 | 08.11.2019 | 2.8.3 | Free | N—A | 2.60 | 4.00 | 3.33 | 1.14 | 2.77 | 4 |
| 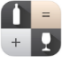 | Alcohol calculator | iOS | 2.5 | N—A | Vladimir Borysiuk. AMM | 13 | 10.02.2016 | 3.2. | Free | N—A | 2.60 | 4.25 | 3.00 | 1.57 | 2.86 | 4 |
| 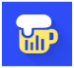 | AlcoTrack | Android | 4.4 | 100000+ | FLX Apps | 1652 | 26.02.2021 | 3.3.5. | Free | N—A | 3.60 | 4.00 | 4.33 | 2.00 | 3.48 | 8 |
| 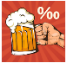 | Alkotester | Android | N—A | 1000+ | uysnon | N—A | 29.01.2020 | 1.0.4 | Free | N—A | 2.00 | 4.75 | 3.33 | 1.29 | 2.84 | 4 |
| 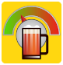 | Virtual AlcoTest | Android | 3.6 | 10000+ | DoubleRouble | 67 | 03.11.2016 | 2.8 | Free | 0.77$ | 2.20 | 4.00 | 3.00 | 2.29 | 2.87 | 5 |
| 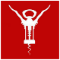 | Alcometer | Android | N—A | 100+ | Rhinocerology | N—A | 13.05.2017 | 1.2 | Free | N—A | 1.80 | 2.25 | 2.33 | 1.29 | 1.92 | 4 |
| 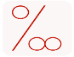 | Alcohol Calculator 2021 | Android | 4.8 | 100+ | Kirov Company | 23 | 10.02.2021 | 1.0.2 | Free | 0.99$ | 2.40 | 4.00 | 3.00 | 1.29 | 2.67 | 4 |
| 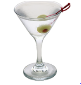 | Alco calculator | Android | 3.7 | 10000+ | One-Eleven Studios | 127 | 02.04.2013 | 0.2 | Free | N—A | 2.60 | 4.00 | 2.00 | 1.29 | 2.47 | 4 |
| 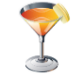 | Alcohol calculator | Android | 3.5 | 1000+ | Aneva Development | 33 | 29.10.2014 | 1.3 | Free | N—A | 2.60 | 4.00 | 2.00 | 1.29 | 2.47 | 4 |
| 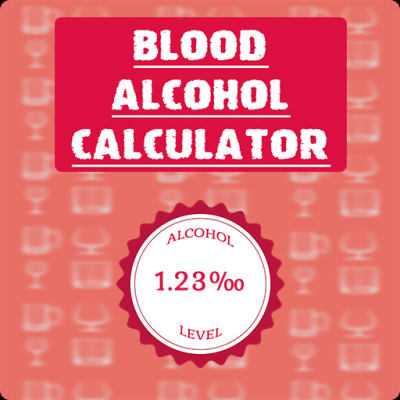 | Blood alcohol calculator | Android | N—A | N—A | N—A | N—A | 16.02.2017 | 1 | Free | N—A | 1.40 | 3.00 | 2.00 | 1.00 | 1.85 | 2 |
| 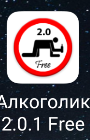 | Alcoholic 2.0.1 Free | Android | N—A | N—A | Riewe | N—A | 04.02.2016 | 2.0.1 | Free | N—A | 1.60 | 3.75 | 3.00 | 1.86 | 2.55 | 3 |
| 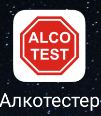 | Alkotester | Android | N—A | N—A | ProntoUfa | N—A | 03.09.2013 | 0.9 | Free | N—A | 1.60 | 3.50 | 2.00 | 1.00 | 2.03 | 2 |
| 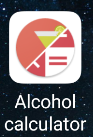 | Alcohol calculator | Android | N—A | N—A | Yurchenko Artyom | N—A | 01.03.2017 | 1.0 | Free | N—A | 1.60 | 3.25 | 2.00 | 1.86 | 2.18 | 4 |
| 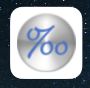 | Calculation promille | Android | N—A | N—A | N—A | N—A | 01.03.2011 | N—A | Free | N—A | 1.40 | 4.00 | 1.00 | 1.14 | 1.89 | 2 |
| 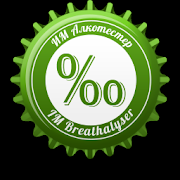 | Alkotester | Android  iOS" | 1.7 | N—A | Andrej Podlubnyj | N—A | 07.12.2016 | 1.0.7 | Free | N—A | 1.60 | 3.25 | 2.00 | 1.14 | 2.00 | 5 |
| 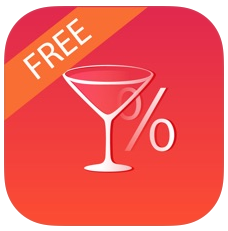 | Alkotester LITE | iOS | 3.8 | N—A | Made mobile creative ou | 203 | 16.12.2016 | 1.0.2 | Free | 2$ | 2.40 | 3.50 | 3.00 | 1.14 | 2.51 | 4 |
| 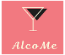 | AlcoMe 1.5 | Android | N—A | 100+ | Andrei Sevastianov | N—A | 01.10.2020 | 1.5 | Free | N—A | 1.80 | 4.00 | 2.67 | 1.29 | 2.44 | 3 |
| 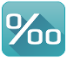 | Alcogram - Сalculator | Android | 4.2 | 1000+ | Rapatundr | 16 | 13.04.2014 | 1.3 | Free | N—A | 1.80 | 4.00 | 2.67 | 1.29 | 2.44 | 4 |
| 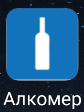 | Alkomer | Android | N—A | N—A | RIA Novosti | N—A | 29.12.2012 | 1 | Free | N—A | 2.40 | 3.75 | 3.67 | 2.29 | 3.03 | 3 |
| **Mobile apps for recording personal alcohol consumption** | | | | | | | | | | | | | | | | |
| 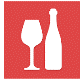 | Alcogram | Android | 4.7 | 100000+ | KursX | 2439 | 24.03.2021 | 2.5 | Free | 1-2$ | 2.40 | 4.00 | 3.33 | 1.57 | 2.83 | 2 |
| 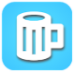 | AlcoTracker | Android | 4.4 | 1000+ | Camilfo | 26 | 06.11.2018 | 0.4 | Free | N—A | 1.60 | 5.00 | 3.33 | 1.14 | 2.77 | 2 |
| 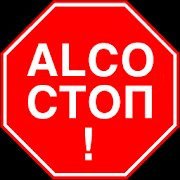 | Alco calculator calendar | Android | 3.4 | 10000+ | Koshkin D&D | 127 | 21.07.2016 | 0.0.7 | Free | N—A | 1.60 | 3.25 | 2.67 | 1.14 | 2.16 | 1 |
| 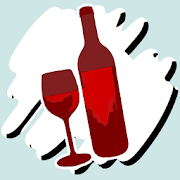 | Alcohol calculator | Android | 4.2 | 5000+ | redpic | 12 | 05.08.2019 | 1.2 | Free | N—A | 2.00 | 3.50 | 2.33 | 1.00 | 2.21 | 3 |
| 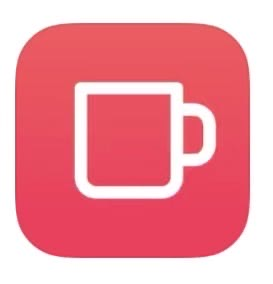 | RedWine Glass | iOS | 4 | N—A | Konstantin Osintsev | 2 | N—A | N—A | Free | N—A | 2.60 | 4.25 | 3.67 | 1.71 | 3.06 | 2 |
| 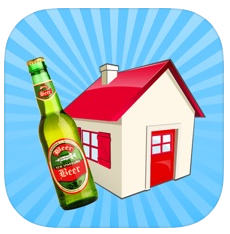 | House of sobriety | iOS | 4.4 | N—A | OU Bamboo Group | 226 | 27.09.2012 | 1.4.1. | Free | N—A | 2.80 | 3.25 | 3.00 | 1.57 | 2.66 | 4 |
| 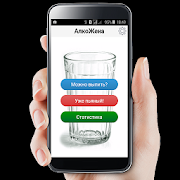 | AlkoWife Free | Android | 3.5 | 500+ | Dmitry Salnikov | 12 | 21.07.2016 | 1.0.0. | Free | N—A | 2.20 | 4.25 | 3.33 | 1.14 | 2.73 | 2 |
| 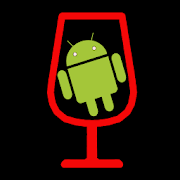 | AlcoDroid Alcohol Tracker | Android | 4.3 | 1000000+ | Myrecek | 5987 | 23.12.2018 | Depends on the device | Free | 2$ | 3.60 | 4.00 | 3.33 | 1.86 | 3.20 | 6 |
| 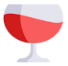 | Alcohol calendar | Android iOS | 3.3 | 10000+ | Alco Apps | 122 | 13.09.2020 | 1.0.2 | Free | 2$-13$ | 3.20 | 4.50 | 4.00 | 1.14 | 3.21 | 5 |
| 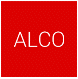 | Alcohol Tracker: Alcohol Calendar | Android | 4.9 | 50+ | Maxim Utyuzhnikov | 7 | 30.12.2020 | 1.0.0 | Free | N—A | 3.00 | 4.00 | 3.00 | 1.86 | 2.96 | 7 |
| 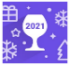 | Drink Days | Android | 4.2 | 5000+ | Hamster Dev | 115 | 06.01.2021 | 1.1.1 | Free | N—A | 2.80 | 3.75 | 4.00 | 1.86 | 3.10 | 7 |
| 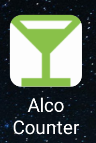 | Alcohol Counter | Android | N—A | N—A | Sidkay | N—A | 17.12.2015 | 1 | Free | N—A | 1.80 | 3.50 | 2.33 | 1.14 | 2.19 | 2 |
| 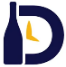 | Drinkky | Android | 5.0 | 5000+ | SD Finance | 8 | 03.03.2021 | 1.0.9 | Free | 0.99$ | 4.00 | 3.25 | 5.00 | 1.86 | 3.53 | 4 |
| 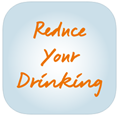 | Reduce your drinking | iOS | 5.0 | N—A | CorporateCom GmbH | 1 | 28.09.2015 | N—A | Free | N—A | 2.80 | 3.25 | 3.00 | 1.57 | 2.66 | 13 |
| 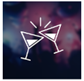 | Wise Drinking | Android iOS | 3.8 | 50000+ | Pernod Ricard SA | 477 | 06.03.2019 | 5.1.0 | Free | N—A | 3.20 | 4.00 | 3.33 | 2.71 | 3.31 | 6 |
| **Mobile apps for screening and brief interventions** | | | | | | | | | | | | | | | | |
| 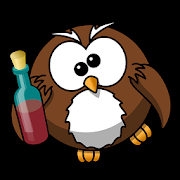 | Alcoholism test | Android | 2.6 | 1000+ | SpeedySnail | 18 | 12.03.2018 | 1.0.4 | Free | N—A | 2.40 | 4.75 | 3.33 | 2.29 | 3.19 | 2 |
| 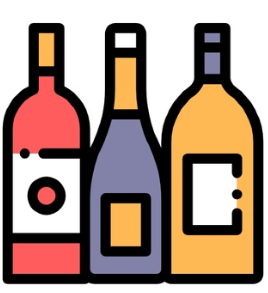 | AUDIT | Android | N—A | N—A | Anna Bunova | N—A | 04.06.2019 | 1.0 | Free | N—A | 2.80 | 4.50 | 4.00 | 3.29 | 3.65 | 5 |
| **Mobile apps for counting the time since last the drinking occasion (sobriety counters)** | | | | | | | | | | | | | | | | |
| 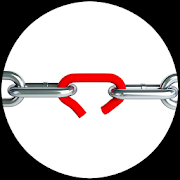 | Freedom | Android | 4.4 | 50000+ | Tikamori | 1451 | 25.02.2021 | 7.1 | Free | 1.30$ | 2.80 | 4.50 | 3.67 | 1.57 | 3.13 | 10 |
| 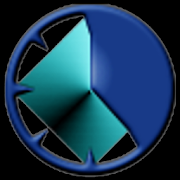 | Net time counter | Android | 4.5 | 500000+ | topotApps | 5793 | 18.02.2013 | 1.1 | Free | N—A | 3.00 | 4.25 | 3.00 | 1.29 | 2.88 | 8 |
| 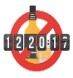 | DWA: Sobriety | Android | N—A | N—A | TC Solution Inc. | 31 | 22.03.2021 | 1.1.0.2 | Free | 1.99$ | 2.60 | 4.00 | 3.33 | 1.71 | 2.91 | 3 |
| 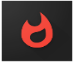 | Break bad habits | Android | 3.9 | 10000+ | Alexander Kolmachikhin | 109 | 23.03.2021 | 11 | Free | 1$ | 2.00 | 4.00 | 2.33 | 1.29 | 2.40 | 7 |
| 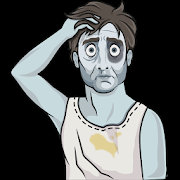 | QUIT DRINKING! | Android  iOS" | 3.5 | 1000+ | Shaft Group | 11 | 03.12.2019 | 1.0 | Free | 3.90$-13$ | 3.80 | 3.75 | 4.00 | 1.71 | 3.32 | 6 |
| 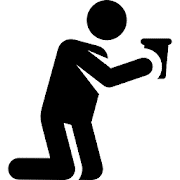 | Stop drinking! | Android | 3.1 | 10000+ | Shaft Group | 78 | 26.01.2019 | 8.2 | Free | N—A | 2.80 | 3.25 | 2.33 | 2.00 | 2.60 | 7 |
| 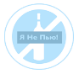 | I do not drink! | Android | 2.3 | 1000+ | M.V.Likhachev | 7 | 09.03.2021 | 1.1.5. | Free | N—A | 2.60 | 3.50 | 2.00 | 1.29 | 2.35 | 1 |
| 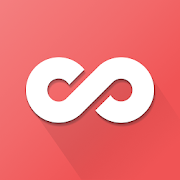 | Quit Addictions and Habits | Android iOS | 4.7 | 1000000+ | despDev | 53516 | 11.03.2020 | 2.0.2 | Free | 2.60$ | 3.20 | 4.50 | 3.33 | 2.57 | 3.40 | 8 |
| **Mobile apps with structured support to reduce alcohol use** | | | | | | | | | | | | | | | | |
| 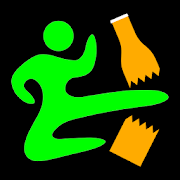 | Stop Drinking (EasyQuit) | Android | 4.9 | 500000+ | Mario Herzberg (Hanna) | 28890 | 19.02.2021 | 1.1.8 | Free | N—A | 4.20 | 4.75 | 3.33 | 3.29 | 3.89 | 15 |
| 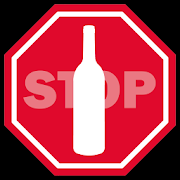 | I do not drink! | Android iOS | 4.8 | 100000+ | А7-studio | 4418 | 25.04.2020 | 1.8 | Free | 1.30$ | 3.40 | 4.50 | 3.67 | 2.57 | 3.53 | 8 |
| 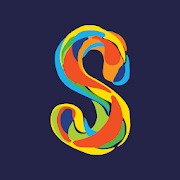 | Sober One - | Android | 4.3 | 1000000+ | Myrecek | 5987 | 23.12.2018 | Depends on the device | Free | 1.90$ | 4.00 | 4.25 | 4.33 | 3.00 | 3.90 | 15 |
| 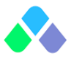 | I Am Sober | Android iOS | 4.9 | 1000000+ | I Am Sober LLC | 24882 | 15.03.2021 | 6.3 | Free | 0.99$ - 74.99$ | 4.20 | 3.75 | 3.67 | 3.00 | 3.65 | 14 |
| **Other mobile apps for reducing alcohol use** | | | | | | | | | | | | | | | | |
| 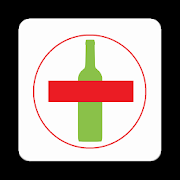 | Quit drinking in one click | Android | 1.8 | 1000+ | OfniCon LLC | 6 | 16.02.2019 | 1.2 | Free | N—A | 1.80 | 4.50 | 1.67 | 1.71 | 2.42 | 4 |
| 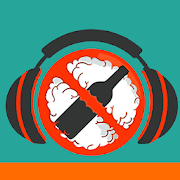 | Hypnosis: Stop drinking while driving | Android | N—A | 100+ | VladTime | N—A | 03.02.2020 | 1.0.5 | Free | N—A | 1.80 | 3.00 | 3.33 | 0.86 | 2.25 | 2 |
| 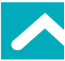 | Sobriety navigator | Android | 3.9 | 5000+ | Arseniy Kaysarov | 50 | 15.07.2019 | 1.0.15 | Free | 6.49$ | 3.00 | 3.75 | 3.33 | 1.00 | 2.77 | 5 |
| 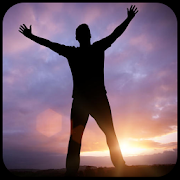 | ControlYourSelf | Android | 4.5 | 10000+ | OSHEMB dev. | 114 | 19.06.2018 | 1.8 | Free | N—A | 2.20 | 3.00 | 2.33 | 1.29 | 2.20 | 4 |
| 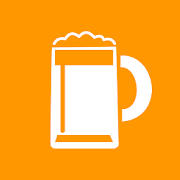 | How much to drink? | Android | 4.5 | 100000+ | XZ Company | 1619 | 18.11.2020 | 1.09 | Free | N—A | 1.80 | 5.00 | 3.67 | 2.43 | 3.22 | 2 |

ABACUS – the App Behavior Change Scale

MARS – the Mobile App Rating Scale

N—A – not available
